# Understanding the interface interaction between U_3_Si_2_ fuel and SiC cladding

**DOI:** 10.1038/s41467-020-16435-x

**Published:** 2020-05-26

**Authors:** Vancho Kocevski, Denise A. Lopes, Antoine J. Claisse, Theodore M. Besmann

**Affiliations:** 1 Nuclear Engineering Program, University of South Carolina, Columbia, SC 29208 USA; 2 Westinghouse Electric Sweden, SE-72163 Västerås, Sweden

**Keywords:** Nuclear fuel, Surfaces, interfaces and thin films, Electronic structure

## Abstract

Triuranium disilicide (U_3_Si_2_) fuel with silicon carbide (SiC) composite cladding is being considered as an advanced concept/accident tolerant fuel for light water reactors thus, understanding their chemical compatibility under operational and accident conditions is paramount. Here we provide a comprehensive view of the interaction between U_3_Si_2_ and SiC by utilizing density functional theory calculations supported by diffusion couple experiments. From the calculated reaction energies, we demonstrate that triuranium pentasilicide (U_3_Si_5_), uranium carbide (UC), U_20_Si_16_C_3_, and uranium silicide (USi) phases can form at the interface. A detailed study of U_3_Si_2_ and SiC defect formation energies of the equilibrated materials yielding the interfacial phases U_20_Si_16_C_3_, U_3_Si_5_ and UC reveal a thermodynamic driving force for generating defects in both fuel and cladding. The absence of either the U_3_Si_2_ or SiC phase, however, causes the defect formation energies in the other phase to be positive, removing the driving force for additional interfacial reactions. The diffusion couple experiments confirm the conclusion with demonstrated restricted formation of U_3_Si_5_, UC, and U_20_Si_16_C_3_/USi phases at the interface. The resulting lack of continuous interaction between the U_3_Si_2_ and SiC, reflects the diminishing driving force for defect formation, demonstrating the substantial stability of this fuel-cladding system.

## Abstract (web summary)

Triuranium disilicide fuel and silicon carbide cladding system is of importance for accident tolerance fuel initiative. Here the authors discuss the role of interface interaction between the U3Si2 fuel and SiC cladding in their use as an advanced concept in light water reactors.

## Introduction

Maintaining the existing high standard of living in many countries, and allowing for improvement in the standard of living for others, will require a significant reliable supply of energy into the future. With the threat of global climate change, providing that energy from sustainable, low-carbon energy sources is at the forefront of energy research. As a mature technology, nuclear energy still remains at the leading edge as a near-term, scalable and low-carbon energy source^1,2^. However, the Fukushima-Daiichi accident raised issues of safety and security for nuclear facilities and materials, and addressing these issues has gained significant support, leading to international accident tolerant fuel (ATF) initiatives^3^. The main goal of an ATF is to provide an improved response to major failures in light water reactors (LWRs) leading to damaging temperatures, such as sustained loss of coolant or overpower-type accidents. The replacement of UO_2_/Zr-alloy fuel was established as a principal goal in improving the accident behavior of the fuel-cladding system, ultimately reducing oxidation kinetics, heat of oxidation in steam, and release of hydrogen^4–6^. Thus, the key to ATF development is replacing the Zr-based cladding with a material less susceptible to steam oxidation and having a lower heat of oxidation. This in turn can require replacing the UO_2_ fuel with higher uranium density phases, at least partially offsetting the increased parasitic neutron adsorption accompanying some of the new cladding concepts.

Due to its known resistance to oxidation and its low parasitic neutron adsorption cross-section, SiC/SiC composites have been considered as an ATF cladding option^6^. Correspondingly, the intermetallic fuel compound U_3_Si_2_ has received particular attention due to its combination of high thermal conductivity, reasonably high melting point, resistance to radiation-induced swelling and amorphization, and moderate oxidation resistance^7^. To assess U_3_Si_2_/SiC composite fuel cladding systems as an ATF option, understanding U_3_Si_2_/SiC interactions and thus their compatibility is paramount. This effort follows on work to refine and better understand phase equilibria and energetics in the U-Si system^8^. A significant contribution to such an assessment can be obtained from computational studies, notably using density functional theory (DFT). DFT has become a valuable tool, complementing experimental findings, providing a more fundamental understanding of observed phenomena, and in some cases, predicting new, useful materials or specific materials’ behavior^9^. However, the potential of DFT for analyzing behavior at interfaces had not been fully recognized until the current efforts, focused on using the calculations to understand the atomistic scale driving forces behind interactions at the U_3_Si_2_/SiC interface. A broader aim of this study is thus to provide direction for using DFT in studying interface interactions among materials in general.

In this study, the phases that can be formed at the U_3_Si_2_/SiC interface were initially established from calculated reaction energies. U_3_Si_2_/SiC interactions were further investigated by computing defect formation energies, using the equilibrium phases determined to form at the U_3_Si_2_/SiC interface. Considering that the interactions are driven by interdiffusion mechanisms enabled by defect formation, the calculated defect formation energies provide a way to understand and help predict the behavior at the atomic scale. The study of the compatibility of U_3_Si_2_ and SiC included exposing U_3_Si_2_ fuel to SiC in diffusion couples at well above likely operating temperatures, resembling severe accident conditions, ensuring interaction, and thus allowing for a thorough characterization of the interfacial zones. In addition, the phases that formed at the U_3_Si_2_/SiC interface were corroborated via characterization of pressed pellets of samples of equimolar U_3_S_2_ and SiC heated to high temperatures.

## Results and discussion

### Interface reaction energies

The first step in understanding U_3_Si_2_/SiC interactions is identifying the phases that can form at the interface by finding the reactions with the most negative energies, Δ*H*_r_. Values of Δ*H*_r_ were calculated using:

1 $$\Delta H_{\mathrm{r}} = \frac{1}{{N_{\mathrm{r}}}}\left( {\mathop {\sum}\limits_i^p {c_i\Delta H_{\mathrm{f}}^i - \mathop {\sum}\limits_j^r {c_j\Delta H_{\mathrm{f}}^j} } } \right)$$

where $\Delta H_{\mathrm{f}}^i$ and $\Delta H_{\mathrm{f}}^j$ are the formation energies of the reactants, *i*, and products, *j*, respectively, and c_*i*_ and c_*j*_ are the reaction coefficients of the products and reactants, respectively; for more details on the formation energies the first-principles calculations section. The sums are over all products, *p*, and all reactants, *r*, and *N*_r_ is the total number of atoms participating in the reaction.

We calculated Δ*H*_r_ values considering three binary and two ternary phases as products: USi, U_3_Si_5_, UC, U_20_Si_16_C_3_, and U_3_Si_2_C_2_. Note that because the U_3_Si_5_ phase has the most negative formation energy in the U–Si system^10,11^, forming higher Si-content phases beyond U_3_Si_5_ would not be energetically favorable, and thus, were not considered. The Δ*H*_r_ of reactions yielding two product phases are shown in Table 1. Reactions generating more than two products result in zero degrees of freedom at fixed temperature and pressure, hence Δ*H*_r_ is not a point, but a continuous line representing a linear combinations of reactions with two products (Supplementary Fig. 1).

**Table 1:** Calculated U_3_Si_2_/SiC interface reaction energies.

#	Reaction	*x*	Δ*H*_r_ (eV/atom)
1	5U_3_Si_2_ + 6SiC = 3U_3_Si_2_C_2_ + 2U_3_Si_5_	0.4545	−0.1300
2	**8U**_**3**_**Si**_**2**_ + **9SiC** **=** **5U**_**3**_**Si**_**5**_ + **9UC**	**0.4706**	**−0.1785**
3	3U_3_Si_2_ + 2SiC = U_3_Si_2_C_2_ + 6USi	0.6000	−0.1202
4	2U_3_Si_2_ + SiC = 5USi + UC	0.6667	−0.1430
5	61U_3_Si_2_ + 27SiC = 9U_20_Si_16_C_3_ + U_3_Si_5_	0.6932	−0.1397
6	**7U**_**3**_**Si**_**2**_ + **3SiC** **=** **U**_**20**_**Si**_**16**_**C**_**3**_ + **USi**	**0.7000**	**−0.1386**

U_3_Si_2_/SiC reaction energies, Δ*H*_r_, listed in increasing molar fraction, *x*, of U_3_Si_2_ in the reaction: *x*U_3_Si_2_ + (1–*x*)SiC. The reactions in bold represent the minimum Δ*H*_r_, i.e., Δ*H*_r_ on the reaction energy convex hull (Supplementary Fig. 1).

The reaction having the most negative Δ*H*_r_, reaction #2, forms U_3_Si_5_ and UC. At higher U_3_Si_2_ to SiC mole ratios, representing the fuel side of the fuel/cladding system, the U_20_Si_16_C_3_ and USi phases form (#6), represented by the minimum Δ*H*_r_ at that composition on the Δ*H*_r_ convex hull (Supplementary Fig. 1). This implies that in addition to U_3_Si_5_ and UC, the pair of phases USi and U_20_Si_16_C_3_ should also exist at the U_3_Si_2_/SiC interface proximate to the fuel surface. Reactions forming U_3_Si_2_C_2_ have significantly more positive Δ*H*_r_ than competing reactions, eliminating its likely presence at the interface.

### Defect formation energies

It is evident from the reaction energies that there is a driving force for interaction between U_3_Si_2_ and SiC. However, phase formation needs atomic transport and accumulation, requiring the generation of defects in U_3_Si_2_ and SiC. The energy for forming a defect, $\Delta E_{\mathrm{f}}^D$, can be evaluated using:

2 $$\Delta E_{\mathrm{f}}^D = E_{{\mathrm{tot}}}^D - E_{{\mathrm{tot}}}^0 - \mathop {\sum}\limits_{i = 1}^N {\Delta N_i^D\left( {\mu _i^0 + \Delta \mu _i} \right)}$$

where $E_{{\mathrm{tot}}}^0$ and $E_{{\mathrm{tot}}}^D$, are the total energies of the supercell without and with a defect, respectively, and $\mu _i^0$ is the standard state chemical potential (DFT calculated energy per atom) of the element *i*. The standard state elements are α-U, diamond-Si and graphite. $\Delta N_i^D$ is the number of atoms of type *i* added ($\Delta N_i^D$ > 0) or removed ($\Delta N_i^D$ < 0) from the perfect supercell to create the defect, and the sum is over all added and removed elements. Δ*μ*_*i*_ is the change in the chemical potential of element *i* resulting from an *N*-phase equilibrium (i.e., from the local environment), which can be calculated by solving the set of linear equations:

3 $$\Delta H_{{\mathrm{f}},k} = \mathop {\sum}\limits_{i = 1}^N {c_{ik}\Delta \mu _i}$$

where the Δ*H*_f*,k*_ is the formation enthalpy of the phase *k*, and *c*_*ik*_ is the mole fraction of element *i* in phase *k*. From Eq. 3 it is evident that to determine the Δ*μ*_*i*_, the number of phases *k* and elements (components) *i* should be equal, as there are zero degrees of freedom at constant temperature and pressure for three stable phases, completely defining the system.

The formation of any defect depends on the chemical potentials (Eq. 2), while the change in the chemical potentials, Δ*μ*_*i*_, in turn, depends on the third phase formed from U_3_Si_2_ and SiC, establishing a 3-phase equilibrium (Eq. 3). In our case, we naturally consider U_3_Si_2_ and SiC to be in equilibrium, hence two phases are already determined, with the candidate third phase being USi, U_3_Si_5_, UC, or U_20_Si_16_C_3_. To model formation of an interface layer that separates the fuel and cladding phases, preventing them from equilibrating with each other, we allowed U_3_Si_2_ or SiC to be replaced with USi, U_3_Si_5_, UC or U_20_Si_16_C_3_. In contrast to the routinely used approach of specifying that two bulk phases are stable^12,13^, our approach allows studying the effect of the formation of a third phase at the interface. This method can also be used as a tool for identifying a phase or phases that could suppress the formation of defects in the phases in contact and hence, prevent interaction.

The calculated defect formation energies in U_3_Si_2_ and SiC from Eq. 2, are detailed in Table 2. The results for U_3_Si_2_-SiC-U_20_Si_16_C_3_ demonstrate that there is a substantial driving force for forming Si defects in U_3_Si_2_; where Si can substitute for U on either of the two U sites and/or occupy one of two interstitial positions. In addition, formation of U vacancies on either U site is favored. Both Si incorporation and formation of U vacancies can lead to the formation of the Si-rich phases USi or U_3_Si_5_. In contrast, equilibrating U_3_Si_2_ and SiC with U_3_Si_5_, UC, or USi, (columns 1, 2, and 3 in Table 2) creates a driving force for inclusion of C on interstitial sites in U_3_Si_2_, potentially forming U_20_Si_16_C_3_. There is also a driving force for Si incorporation on the 2b site when U_3_Si_5_ and UC are present, and Si on the U1 anti-site when UC is present. The negative defect formation energies when either of the three phases U_3_Si_5_, UC, and U_20_Si_16_C_3_ are present implies that there is a substantial driving force for the formation of the Si-rich phase U_3_Si_5_.

**Table 2:** Calculated defect formation energies in U_3_Si_2_ and SiC.

	Three-phase equilibria					
Point defect in U_3_Si_2_	U_3_Si_2_-SiC-U_3_Si_5_	U_3_Si_2_-SiC-UC	U_3_Si_2_-SiC-USi	U_3_Si_2_-SiC-U_20_Si_16_C_3_	U_3_Si_2_-U_3_Si_5_-U_20_Si_16_C_3_*	U_3_Si_2_-U_3_Si_5_-UC*
U1 vac.	0.87	0.41	1.10	**−10.27**	0.87	0.87
U2 vac.	2.22	1.76	2.44	**−8.93**	2.22	2.22
Si vac.	1.13	1.82	0.79	17.84	1.13	1.13
U-on-Si	0.66	1.81	0.10	28.52	0.66	0.66
Si-on-U1	0.40	**−0.75**	0.97	**−27.45**	0.40	0.40
Si-on-U2	1.67	0.52	2.23	**−26.19**	1.67	1.67
Si in site 2b	**−0.24**	**−0.93**	0.10	**−16.95**	**−0.24**	**−0.24**
Si in site 4h	1.63	0.94	1.97	**−15.09**	1.63	1.63
C-on-U1	1.29	1.52	1.18	6.86	3.15	2.44
C-on-U2	2.27	2.50	2.15	7.84	4.12	3.42
C-on-Si	1.27	2.65	0.59	34.70	3.12	2.42
C in site 2b	**−1.29**	**−0.60**	**−1.63**	15.42	0.56	**−0.14**
C in site 4g	0.06	0.75	**−0.28**	16.78	1.92	1.21
C in site 4h	**−0.24**	0.45	**−0.58**	16.48	1.62	0.91
C in site 8i	6.42	7.11	6.08	23.14	8.28	7.57

Point defect in SiC	U_3_Si_2_-SiC-U_3_Si_5_	U_3_Si_2_-SiC-UC	U_3_Si_2_-SiC-USi	U_3_Si_2_-SiC-U_20_Si_16_C_3_	SiC-UC-U_20_Si_16_C_3_*	SiC-U_3_Si_5_-UC*
Si vac.	7.25	7.95	6.92	23.97	7.78	7.69
C vac.	4.82	4.13	5.16	**−11.89**	4.30	4.39
Si vac. in Si-on-C**	**−0.05**	0.64	**−0.39**	16.67	0.48	0.38
U-on-Si	4.34	5.49	3.78	32.20	5.49	5.49
U-on-C	11.18	10.95	11.29	5.61	11.27	11.47
Si-on-C	4.87	3.49	5.55	**−28.56**	3.81	4.01
C-on-Si	2.48	3.86	1.81	35.91	3.54	3.35
U in site 4b	12.88	13.34	12.66	24.03	13.50	13.60
U in site 4d	14.61	15.07	14.39	25.76	15.23	15.33

Defect formation energies ($\Delta E_{\mathrm{f}}^D$) in eV/defect atom, in U_3_Si_2_ at chemical potentials governed by the indicated three-phase equilibria. Negative $\Delta E_{\mathrm{f}}^D$ values are shown bold.

^*^Fuel and cladding not in contact.

**Si-on-C anti-site with vacancy on Si anti-site.

The formation of Si vacancies was found to be not energetically favored for all studied 3-phase equilibria, thus U_3_Si_2_ will not decompose in contact with SiC to form a more U-rich phase. The formation energy for C interstitials is significantly negative, however, yet unlike Si, C cannot form anti-site defects in U_3_Si_2_. Overall, there is a much higher preference for Si forming a U-Si-C ternary phase such as U_20_Si_16_C_3_, as opposed to a C-rich phases such as U_3_Si_2_C_2_. This conclusion agrees with the computed reaction energies that imply that U_3_Si_2_C_2_ will not form at the interface (Table 1).

SiC in contrast, is understood to not tolerate Frenkel or Schottky defects, yet does display a wide variety of polymorphs based on stacking faults, which is again demonstrated in the positive incorporation and vacancy formation energies computed here (Supplementary Tables 2 and 3). Thus the U_3_Si_2_-SiC-U_20_Si_16_C_3_ phase equilibrium-generated driving force for generating C vacancies in SiC, as well as Si-on-C anti-site defects, implies SiC decomposition. Decomposition product C will react with U_3_Si_2_, promoted by the equilibria, forming UC. Formation of the favored Si vacancy on a Si-on-C anti-site, as well as the driving force for forming Si vacancies, become the mechanism for forming a more Si-rich phase proximate to U_3_Si_2_. Ultimately, the net effect is the surface degradation of SiC at the contact interface.

Once a phase is formed on the fuel side such that U_3_Si_2_ and SiC can no longer directly interact, the governing three-phase equilibrium becomes either U_3_Si_2_-U_3_Si_5_-U_20_Si_16_C_3_ or U_3_Si_2_-U_3_Si_5_-UC (columns 6 and 7 in Table 2). Under such conditions the defects have positive or only slightly negative formation energies interrupting any further interactions. The small negative value for Si on the 2b interstitial site allows diffusion of Si in U_3_Si_2_, promoting the formation of U_3_Si_5_. When U_3_Si_2_ is replaced in the equilibria by UC, generating the SiC-UC-U_20_Si_16_C_3_ or SiC-UC-U_3_Si_5_ phase equilibria, defect formation energies are positive. Substituting USi for U_3_Si_5_ or U_20_Si_16_C_3_, causes the considered defects to also have positive formation energies (results not shown), and were thus not considered. The implications of the defect formation energy calculations for the various equilibria are that after initial reactions between U_3_Si_2_ and SiC yielding interface phases, further interdiffusion would be very slow, essentially halting any further interactions. The only exception could be some transport of Si in U_3_Si_2_.

The defect formation energies suggest that when any of the three interface phases, U_3_Si_5_, UC, or U_20_Si_16_C_3_ are present, there is a driving force for forming defects in both U_3_Si_2_ and SiC. On the other hand, the formation of any of these third phases requires a critical number of atoms to interact, the source of which are solely the formation of vacancies in U_3_Si_2_ and SiC. However, creating any vacancies, especially in SiC, requires substantial energy, such as high temperatures, thus serving as the bottleneck for initializing the U_3_Si_2_/SiC interaction. An analysis of the kinetics of the atoms at the U_3_Si_2_/SiC interface, such as their diffusivity over the interface, would provide a more thorough understanding of the influence of the temperature on the U_3_Si_2_/SiC interaction. In addition, the most complete picture of the driving force for U_3_Si_2_/SiC interactions can be obtained by looking at the defect formation energy at different types of U_3_Si_2_ and SiC surfaces forming a U_3_Si_2_/SiC interface. However, because the U_3_Si_2_ and SiC surfaces at the interface are not clearly defined, several possible interface combinations need to be considered, yet analysis of the entire number of possible defects would be prohibitive.

The results show that U_20_Si_16_C_3_ phase encourages formation of U vacancies and Si incorporation in U_3_Si_2_ yielding U_3_Si_5_, on the fuel side. The ternary phase also promotes carbon vacancy formation and Si-on-C anti-site occupancy in otherwise very inert SiC, causing its decomposition and the formation of UC on the surface of the SiC. In turn, the U_3_Si_5_ and UC phases promote formation of Si and C vacancies in SiC and C interstitials in U_3_Si_2_, further supporting the formation of all three interface phases. When the U_3_Si_2_ and SiC are no longer in equilibrium due to an interface layer, there is no longer a driving force for most of the defects, and thus there is little observable interaction between the fuel and cladding, with the exception of some formation of U_3_Si_5_ in the fuel.

### Diffusion couple experiments

**Fig. 1:**
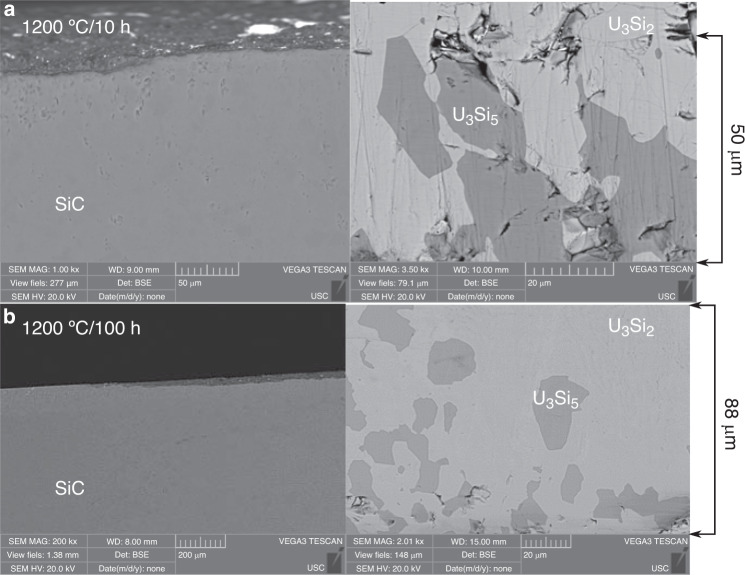
SEM analysis of U_3_Si_2_ and SiC. Polished cross-section of the U_3_Si_2_ and SiC surfaces after exposure at 1200 °C for: (**a**) 10 h, and (**b**) 100 h. The depth of Si diffusion into the U_3_Si_2_ can be seen in the formation of U_3_Si_5_ well below the surface.

The computational results are reflected in the experimental observations for the U_3_Si_2_/SiC system diffusion couples annealed at 1200 °C/10 h and 1200 °C/100 h. The microstructures are shown in Fig. 1, where samples annealed at 1000 °C/100 h indicate no observable reaction, reflecting the results of differential scanning calorimetry (DSC) on the phase mixture (Supplementary Fig. 2). While the materials of the 1200 °C couples also did not adhere, easily separating during the disassembly of the jigs, an interdiffusion layer at the U_3_Si_2_ surface was observed with proximate Si-rich grains (Fig. 1a, b), reflecting the defect formation energies that allow incorporation of Si in U_3_Si_2_. The longer exposure time sample displayed U_3_Si_5_ grains beyond the interdiffusion layer (Fig. 1b), indicating diffusion of Si in the U_3_Si_2_. This would naturally be the result of the calculated driving force for Si incorporation in U_3_Si_2_ occuring regardless of whether the U_3_Si_2_ and SiC are in contact.

**Fig. 2:**
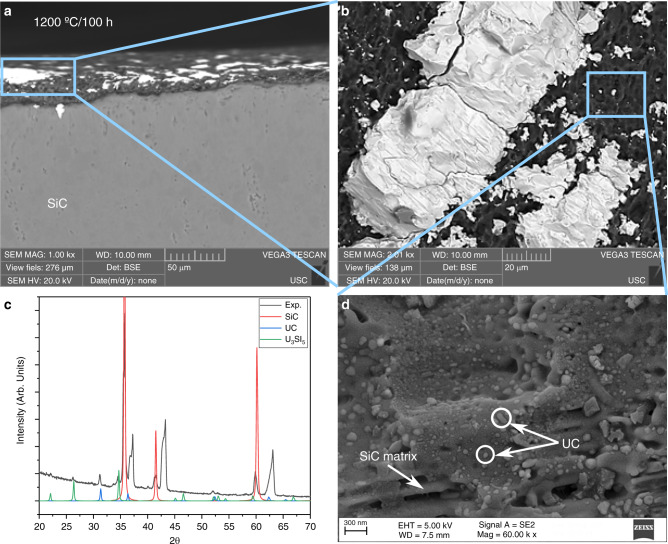
Analysis of SiC. (**a**–**b**, **d**) SiC surface SEM images, and (**c**) low angle XRD, for a diffusion couple sample annealed at 1200 °C/100 h.

No change in SiC composition was observed, with surface reactions causing some decomposition thus a concavity. SiC is relatively resistant to interdiffusing elements, and the observation reinforces the computed conclusion that the driving force for forming Si and C vacancies in SiC essentially decompose the surface. Microscopy images of the SiC surface and results of low angle x-ray diffraction (XRD) are seen in Fig. 2 where two different morphologies are observed: (i) large particles with a faceted surface (10–100 μm), typical of intermetallics such as U_3_Si_2_, and (ii) fine, dispersed particles, mainly found in cavities in the SiC surface. Both phases are uranium compounds as indicated by the bright contrast in the scanning electron microscopy (SEM) images. The larger particles were analyzed by energy dispersive spectroscopy (EDS) and had an average atomic composition of 52.7% (±2.8) U and 47.3% (±2.7) Si. This composition is close to USi, but it can also indicate the ternary U_20_Si_16_C_3_ phase, as the low atomic number of C prevents it from being properly detected by EDS, and formation of either USi or U_20_Si_16_C_3_ were shown to be possible. The fine, dispersed particles were too small to be analyzed by EDS, but low angle XRD yielded peaks that could be ascribed to β-SiC, U_3_Si_5_, and UC (Fig. 2c). The similar fcc structures of UC and SiC could encourage UC formation on the SiC surface, although their lattice parameters are markedly dissimilar. To further investigate the nature of these particles, high resolution SEM images were obtained (Fig. 2d) revealing the presence of spherical features adhered to the SiC surface. The spherical shape could reflect coherent growth on the SiC surface^14^. In addition, the phase is seen to be U-rich based on the bright contrast in the backscatter electron image.

To benchmark phase formation in the U_3_Si_2_-SiC system, annealed 1:1 molar mixtures of U_3_Si_2_:SiC powders were analyzed using DSC. The thermal trace of Supplementary Fig. 2a indicates an exothermic reaction occurring at ~1135 °C, supporting the lack of interactions observed in the diffusion couples annealed at 1000 °C. XRD analysis before the heating cycle showed major peaks for U_3_Si_2_ and SiC, with a minor amount of USi (FeB-type structure), the latter likely the result of the arc-melting synthesis of the original U_3_Si_2_ material. After heat treatment the XRD spectra indicate the presence of U_3_Si_5_, UC, and a minor amount of U_20_Si_16_C_3_ (~6 mol%), in agreement with the reported U-Si-C phase diagram 11^15^ and the computed reaction energies (Table 1). The results all suggest that the larger particles on the SiC surface are indeed the U_20_Si_16_C_3_ phase (Fig. 2b).

In conclusion, a detailed investigation of the interaction between U_3_Si_2_ and SiC using DFT calculations of potential reactions and defect formation energies supported by experiment has delineated likely fuel-cladding interactions. Formation of U_3_Si_5_ and UC at the interface was shown to be favored, as was formation of U_20_Si_16_C_3_ and USi on the U_3_Si_2_ fuel side. These phases allow formation of complementary defects in both U_3_Si_2_ and SiC, driving the interaction between the phases. Once an interaction phase is interposed between U_3_Si_2_ and SiC, the governing three-phase equilibrium becomes either U_3_Si_2_-U_3_Si_5_-U_20_Si_16_C_3_ or U_3_Si_2_-U_3_Si_5_-UC. Under such conditions the defects have positive or only slightly negative formation energies interrupting any further interactions. The small negative energy value for Si on the 2b interstitial site allows diffusion of Si in U_3_Si_2_, promoting the formation of U_3_Si_5_, with defect formation energies becoming positive when U_3_Si_2_ is replaced by UC. The major implication of the defect formation energy calculations for the various equilibria are that after initial reactions between U_3_Si_2_ and SiC yielding interface phases, further interdiffusion becomes very slow, essentially halting any further interactions.

With regard to temperature dependence, the described interactions require formation of vacancies in U_3_Si_2_ and SiC, which need sufficient energy for their diffusion, explaining the absence of any observed interaction in the diffusion couples at 1000 °C. At the higher temperature of 1200 °C, U_3_Si_5_, UC, U_20_Si_16_C_3_, and USi phases were observed at the interface, a result of limited interdiffusion, as implied by the DFT analysis. Thermal analysis of U_3_Si_2_:SiC equimolar mixtures indicated that U_20_Si_16_C_3_ is stable in the phase region rather than USi. The cessation of fuel-cladding interactions due to the formation of a region devoid of either U_3_Si_2_ and SiC even at significantly elevated temperatures, argues for a very stable U_3_Si_2_ fuel/SiC cladding system and thus a potentially valuable ATF candidate.

## Methods

### First-principles calculations

DFT calculations were performed with the Vienna Ab initio Simulation Package (VASP)^16,17^. The electron exchange correlation was modeled using the generalized gradient approximation (GGA) of Perdew, Burke and Ernzernhof (PBE)^18^ and projector augmented wave (PAW) potentials^19,20^. To better describe the correlated nature of the U 5*f* electrons, we used the DFT + *U* method with Dudarev’s rotationally invariant approach^21^ and an effective *U*, *U*_eff _= 1.5 eV (*U*_eff_ = *U* – *J*, *U* = 1.5 eV and *J* = 0.0 eV). The unit cells for U_3_Si_2_ and SiC were fully relaxed using a cut-off energy of 600 eV to expand the electronic wave functions. Convergence criteria of 0.01 eV/Å^−1^ and 10^−4^ eV was adopted for the forces and total energy, respectively. We used 2 × 2 × 4 and 3 × 3 × 3 supercells of U_3_Si_2_ and SiC, respectively for calculating the defect formation energies. A γ-centered Monkhorst-pack k-point spacing of <0.02 Å^−1^ was applied for sampling the Brillouin zone for each structure.

### Point defects

**Fig. 3:**
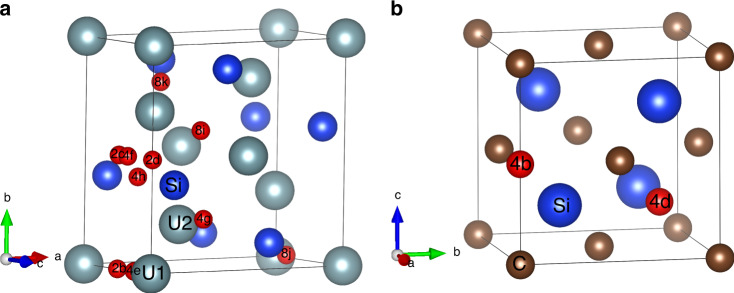
Position of interstitial sites in U_3_Si_2_ and SiC. Interstitial sites (red spheres) in (**a**) U_3_Si_2_, and (**b**) SiC treated as point defects in DFT calculations. The U, Si, and C atoms are shown as gray, blue, and brown, respectively.

The fuel/cladding interaction mechanism was further evaluated by calculating the formation energies of point defects in U_3_Si_2_ and SiC. The incorporation of C and Si in U_3_Si_2_ was modeled considering the two U sites, U1 (2a) and U2 (4h), the Si site, and 10 interstitials sites with Wyckoff positions: 2b, 2c, 2d, 4e, 4f, 4g, 4h, 8i, 8j, and 8k (Supplementary Fig. 3a and Table 1). Of the 10 interstitial sites, Si relaxed to only two sites 2b and 4h, while C relaxed to four sites 2b, 4g, 4h, and 8i. The incorporation of U and Si in SiC were modeled considering the Si and C sites, and two interstitial sites with Wyckoff positions 4b (0, 0, 0.5) and 4d (0.75, 0.75, 0.25) (Fig. 3b). Vacancies on U and Si sites in U_3_Si_2_, and Si and C vacancies in SiC were also considered.

Obtaining optimal total energies require using a *U*_eff_ value that most appropriately represents the structure and the electron correlation in a system. For example, the properties of α-U are best represented by using only GGA^22^, i.e., *U*_eff _= 0 eV, while it has been shown that the lowest *U*_eff_ that best represent U_3_Si_2_ phonons is *U*_eff _= 1.5 eV^23^. However, because of the use of different *U*_eff_ values, the resulting Δ*H*_f_ values cannot be directly compared. Therefore, to allow consistent comparison of Δ*H*_f_ for all phases, the methodology for correcting Δ*H*_f_ values proposed by Jain et al.^24^ was applied, using experimentally reported formation energies^25^. Shown in Table 3 are the corrected formation energies. For defects where uranium is considered as potentially incorporated into SiC, an on-site correlation with *U*_eff _= 1.5 eV was applied.

**Table 3:** Corrected formation energies of the studied phases.

Phase	Space group	Calculated Δ*H*_f_	Experimental Δ*H*_f_	Difference (calc. – exp.)	Calculation method
SiC	F-43m (216)	−0.2063	−0.3465	−0.1402	GGA
U_3_Si_2_	P4/mbm (127)	−0.3298	−0.3538	−0.0240	GGA+*U*
USi	Pnma (62)	−0.4535	−0.4335	0.0200	GGA+*U*
U_3_Si_5_*	P6/mmm (191)	−0.4808	−0.4480	0.0328	GGA+*U*
UC	Fm-3m (225)	−0.4459	−0.5055	−0.0596	GGA+*U*
U_20_Si_16_C_3_	Cmmm (65)	−0.4502	−0.4450	0.0052	GGA+*U*
U_3_Si_2_C_2_	I4/mmm (139)	−0.3733		0.3733	GGA+*U*

Calculated formation enthalpies, Δ*H*_f_, in eV/atom, of U-Si-C binary and ternary compounds used for calculating Δ*H*_r_ and Δ*μ*_i_, compared to tabulated values^25^.

*The U_3_Si_5_ structure was taken from our previous cluster expansion study^11^.

## Experiments

### Materials

The U_3_Si_2_ material for the current effort was prepared from depleted uranium (Los Alamos National Laboratory, 99.98%) and elemental silicon (Sigma Aldrich, 99.99%) by arc-melting^26^. Arc-melting was conducted inside a glovebox maintained at an oxygen and H_2_O level <0.1 ppm. In addition, an in-line getter reduced the argon arc-melter purge gas to 10^−10^ ppm oxygen. The samples were arc-melted 5–6 times to ensure homogeneity. Chemical vapor deposition (CVD) SiC (β-phase) (Morgan Advanced Ceramics, Ultra-pure 99.9995%) was used in the diffusion couples as being representative of SiC-SiC composites as these are expected to be produced via chemical vapor infiltration (CVI) and thus will have a CVD SiC surface in contact with the fuel. For the experiments using mixed U_3_Si_2_-SiC powders, a commercial β-SiC powder (Alfa Aesar, 99.8% purity) was used with reported particle size of ~1 μm and surface area of a 11.5 m^2^/g. The U_3_Si_2_ powder was prepared by grinding in a tool steel mortar and pestle.

### Diffusion couples

**Fig. 4:**
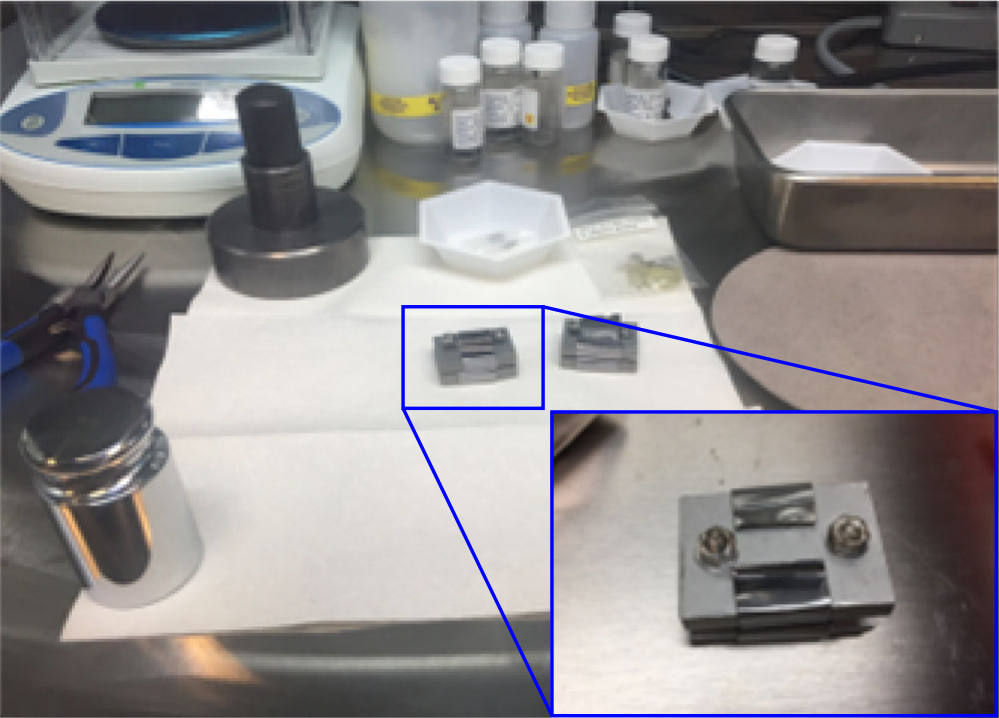
Diffusion couple setup. Diffusion couple fixed in a molybdenum-jig inside a glovebox.

Sections ~2 mm in thickness of U_3_Si_2_, and ~1 mm in thickness of the CVD SiC were cut using a precision, diamond blade saw. The U_3_Si_2_ sample was ground and polished, using #600 and #1200 mesh silicon carbide paper. The CVD SiC was ground using diamond lapping plates of #600, #1200, #1800 mesh. Subsequently, both samples were sequentially polished using 9 μm, 3 μm, 1 μm, and 0.25 μm diamond suspensions, with the final two polishing steps performed inside of a controlled atmosphere (<1 ppm H_2_O and <0.1 ppm O_2_) glovebox to minimize the formation of an oxide layer. The U_3_Si_2_/SiC diffusion couple was assembled in the glovebox using a molybdenum jig (Fig. 4). Tantalum foil was interposed between the molybdenum plates and the samples to avoid potential reactions and the jig was wrapped in tantalum foil to getter residual oxygen. Immediately after assembling, the samples were transferred to a controlled atmosphere, resistance-heated tube furnace (CM 1730-12HT). The high temperature exposures were carried out for 10 or 100 h in flowing argon purified using a GEN’Air oxygen pump (SETNAG) to reduce the gas to 10^−10^ ppm O_2_.

### DSC analysis

The DSC samples were a 1:1 molar ratio mixture of U_3_Si_2_ and β-SiC powders as described above, with samples of ~1 g were manually blended and pressed into pellets. The studies were performed in a Netzsch STA-409 simultaneous thermal analyzer. The samples were heated to 1400 °C for 2 h at 5 °C/min under flowing purified argon, as previously described for the diffusion couples. The system was used in DSC mode and the thermal cycle was repeated twice to obtain a baseline for correction and detection of the onset of reactions. Characterization included SEM with phase composition determined by EDS using a Tescan Vega 3 SEM and a Zeiss Ultra plus field emission SEM (FESEM). Powder XRD analysis was carried out using a Rigaku Ultima IV instrument with Cu-Kα radiation and a scan over 20–100° at 0.02°/s. Rietveld analyses was performed using MAUD software^27,28^.

## Supplementary information

Supplementary Information

Peer Review File

## Data Availability

The authors declare that the data supporting the findings of this study are available within the paper and its supplementary information files. Additional data that support the findings of this study are also available from the corresponding author upon reasonable request and archived on Open Science Forum https://osf.io/mdkzr/.
