# Supplementary material for: Understanding the interface interaction between U3Si2 fuel and SiC cladding
Source: Nat Commun. 2020 May 26;11:2621. doi: 10.1038/s41467-020-16435-x (PMC7250824; doi:10.1038/s41467-020-16435-x)
Supplement: Supplementary file 1 — Supplementary Information [file 41467_2020_16435_MOESM1_ESM.pdf]

# Supporting Information

## Understanding the interface interaction between $U_3Si_2$ fuel and SiC based cladding

Vancho Kocevski[1,*], Denise L. Adorno[1,2], Antoine J. Claisse[2], Theodore M. Besmann[1]

[1]*Nuclear Engineering Program, University of South Carolina, Columbia SC 29208*
[2]*Westinghouse Electric Sweden, SE-72163, Västerås, Sweden*
**Corresponding author; email: vancho.vk@gmail.com.*

### Reaction Energies

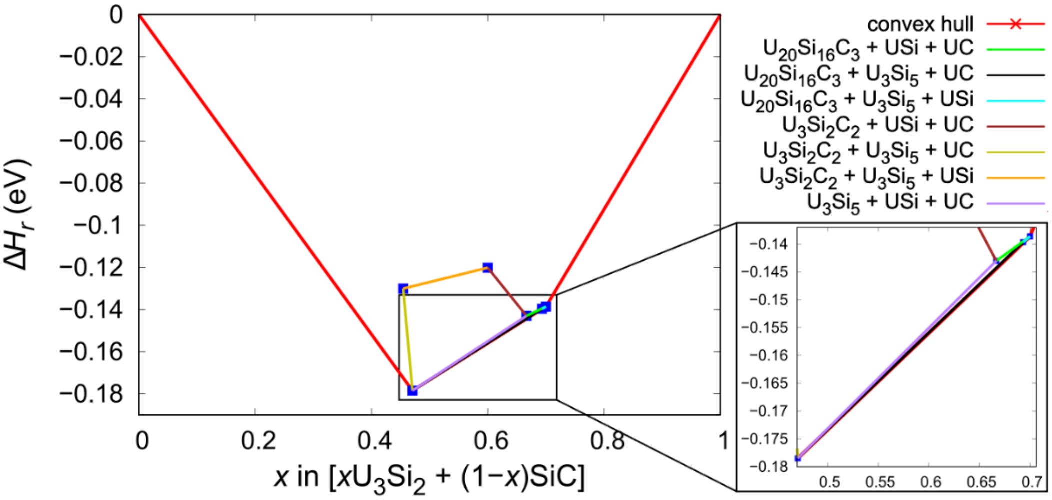


**Figure S1. $U_3Si_2$/SiC interface reaction energies and convex hull for reactions with 3 products.** $U_3Si_2$/SiC interface reaction energies, in eV/atom, as a function of the molar fraction, $x$, of $U_3Si_2$ in the reaction: $xU_3Si_2$ + (1–$x$)SiC. The energies of the reactions with two products are shown in blue points, and for the reactions with three products are shown with lines.

### Interstitial Sites

**Table S1. $U_3Si_2$ and SiC Interstitial site positions and their stability.** List of interstitial sites in $U_3Si_2$ with their Wyckoff position and coordinates, noted as stable (Y) or not stable (N) after relaxation. In the cases where the defect relaxed to a different position, the fractional coordinates of the relaxed site are given.

| # | Position | Fractional Coordinates | Stable |
|---|---|---|---|
| 1 | 2b | (0.00, 0.00, 0.50) | Y |
| 2 | 2c | (0.00, 0.50, 0.50) | N (relaxed to 4h) |
| 3 | 2d | (0.00, 0.50, 0.00) | N (relaxed to 4g) |
| 4 | 4e | (0.00, 0.00, 0.75) | N (relaxed to 2b) |
| 5 | 4f | (0.00, 0.50, 0.63) | N (relaxed to 4h) |
| 6 | 4g | (0.30, 0.20, 0.00) | Y (only for C) |
| 7 | 4h | (0.12, 0.38, 0.50) | Y |
| 8 | 8i | (0.30, 0.50, 0.00) | Y (only for C) |
| 9 | 8j | (0.88, 0.03, 0.50) | N (relaxed to 2b) |

| | | | |
|---|---|---|---|
| 10 | 8k | (0.30, 0.80, 0.25) | N |

## Incorporation Energies

The incorporation energy, $\Delta E_{\mathrm{inc}}$, for an element occupying a vacant lattice site is calculated using:

$$\Delta E_{\mathrm{inc}} = E_{\mathrm{tot}}^{D} + \Delta E_{j}^{\mathrm{vac}} - E_{\mathrm{tot}}^{\mathrm{vac}} - \mu_{i}^{0} \quad \text{(S1)}$$

where $E_{\mathrm{tot}}^{D}$, and $E_{\mathrm{tot}}^{\mathrm{vac}}$, are the DFT computed total energies of the supercells with a substitutional defect, and with a defect site vacancy, respectively. $\mu_{i}^{0}$ the chemical potential (total energy) of the standard state elemental species *i,* here α-U, C as graphite, and Si in the diamond structure. $\Delta E_{j}^{\mathrm{vac}}$ is the energy required to create a vacancy on site *j*, calculated using:

$$\Delta E_{j}^{\mathrm{vac}} = E_{\mathrm{tot}}^{vac} - E_{\mathrm{tot}}^{0} - \mu_{j}^{0} \quad \text{(S2)}$$

where $E_{\mathrm{tot}}^{0}$ is the total energy of a supercell without a defect. $\Delta E_{j}^{\mathrm{vac}} = 0$ in the case when an element is incorporated in an interstitial site.

**Table S2. Energies for incorporating atoms in $U_3Si_2$ and SiC.** Calculated incorporation energies, $\Delta E_{\mathrm{inc}}$, of Si and C in $U_3Si_2$; and U and Si in SiC. The references states used are the chemical potential of α-U, SiC (fcc) and diamond structure Si.

| Type of point defect in $U_3Si_2$ | $\Delta E_{\mathrm{inc}}$ (eV) |
|---|---|
| Si in U1 vacancy | **-0.27** |
| Si in U2 vacancy | 1.00 |
| Si interstitial site 2b | **-0.97** |
| Si interstitial site 4h | 0.90 |
| C in U1 vacancy | 1.67 |
| C in U2 vacancy | 2.65 |
| C in Si vacancy | 2.32 |
| C interstitial site 2b | **-0.97** |
| C interstitial site 4g | 0.38 |
| C interstitial site 4h | 0.08 |
| C interstitial site 4f | 6.74 |
| **Type of point defect in SiC** | **$\Delta E_{\mathrm{inc}}$ (eV)** |
| U in Si vacancy | 5.01 |
| U in C vacancy | 10.80 |
| Si in C vacancy | 3.82 |
| C in Si vacancy | 3.54 |
| U interstitial site 4b | 12.82 |
| U interstitial site 4d | 14.55 |
| Si interstitial site 4b | 4.87 |
| Si interstitial site 4d | 8.52 |

**Table S3. Vacancy formation energies in $U_3Si_2$ and SiC.** Calculated vacancy formation energies, $\Delta E_j^{\mathrm{vac}}$, in $U_3Si_2$ and SiC.

| Vacancies in $U_3Si_2$ | $\Delta E_j^{\mathrm{vac}}$(eV) |
|---|---|
| U1 | 0.93 |
| U2 | 2.28 |
| Si | 1.86 |
| Vacancies in SiC | $\Delta E_j^{\mathrm{vac}}$(eV) |
| Si | 7.99 |
| C | 5.50 |

## Equimolar Reaction

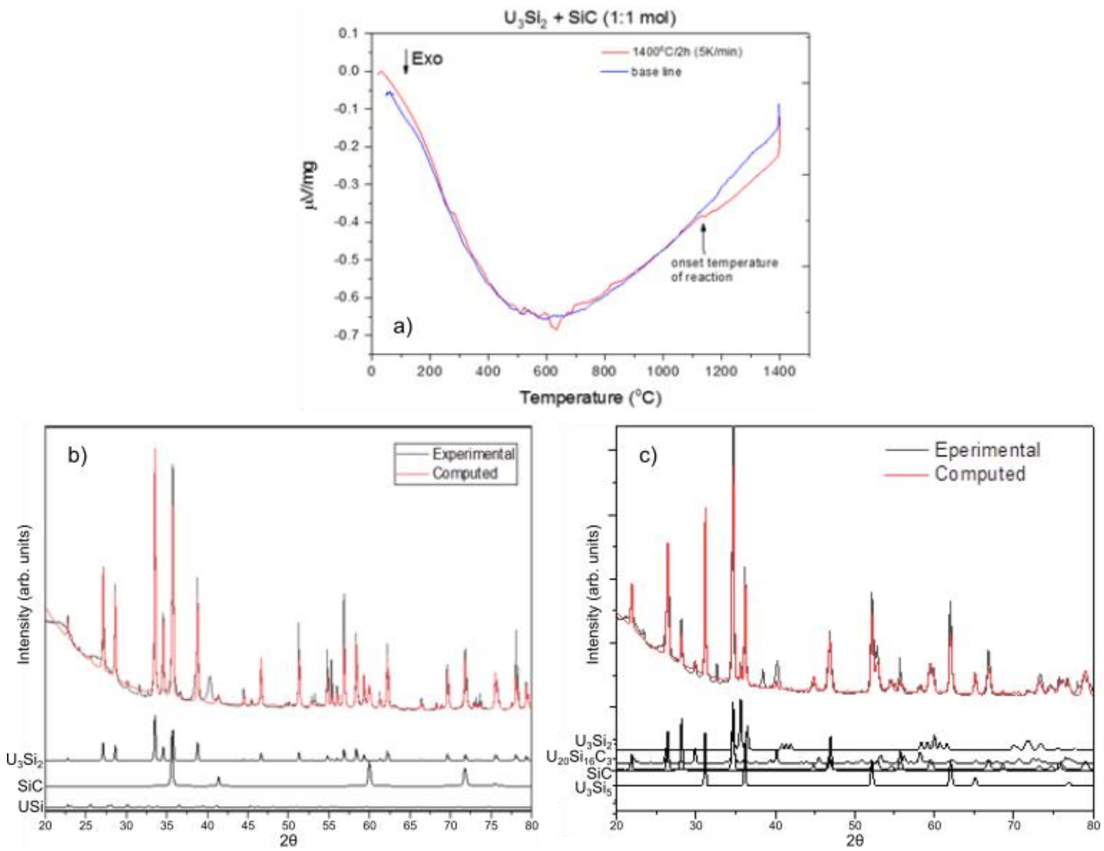


**Figure S2. Analysis of $U_3Si_2$:SiC equimolar mixture.** (a) DSC heating profile of $U_3Si_2$:SiC (1:1 mol) sample as a function of temperature; (b) Rietveld refined XRD pattern for the sample before; and (c) after thermal cycling.
